# Identifying Metabolic Pathways That Drive Gestational Diabetes Mellitus: Protocol for a Prospective Observational Study

**DOI:** 10.2196/92064

**Published:** 2026-11-11

**Authors:** Felix Jebasingh, Lakshmi Marri Harika, Shajith Anoop, Mandisa Christian, Sherin Daniel, Santhosh Joseph Benjamin, Swati Rathore, Reeta Vijaya Selvi, Manisha Madhai Beck, Anuja Abraham, RN Preethi, Ullas Kolthur-Seetharam, Mahendra Sonawane, Sarah Price, Elif Ilhan Ekinci, Nihal Thomas

**Affiliations:** 1 Department of Medicine, The University of Melbourne, Parkville, Victoria, Australia; 2 Department of Endocrinology, Diabetes and Metabolism, Christian Medical College Vellore, 810, Ida Scudder Road, Vellore, Tamil Nadu, 632004, India, 91 416-2282181; 3 Department of Pathology, Christian Medical College Vellore, Vellore, Tamil Nadu, India; 4 Department of Obstetrics and Gynaecology, Christian Medical College Vellore, Vellore, Tamil Nadu, India; 5 Department of Biological Sciences, Tata Institute of Fundamental Research, Hyderabad, Telangana, India; 6 Department of Biological Sciences, Tata Institute of Fundamental Research, Mumbai, Maharashtra, India; 7 Centre for DNA Fingerprinting & Diagnostics (CDFD), Hyderabad, Telangana, India; 8 Department of Obstetric Medicine, Royal Women's Hospital, North Melbourne, Victoria, Australia; 9 The Australian Centre for Accelerating Diabetes Innovation (ACADI), Melbourne Medical School, University of Melbourne, Melbourne, Victoria, Australia; 10 Centre for Stem Cell Research (A Unit of inStem, Bengaluru), Christian Medical College Vellore, Vellore, Tamil Nadu, India

**Keywords:** gestational diabetes mellitus, metabolomics, placenta, metabolites, metabolic pathways

## Abstract

**Background:**

Gestational diabetes mellitus (GDM) is a disorder characterized by hyperglycemia that is first recognized during pregnancy due to a mismatch between placental insulin production and increased maternal need, exacerbating insulin resistance. Few studies have systematically examined the metabolic markers and pathways underlying GDM from diagnosis through the postnatal period, including placental metabolic changes through metabolomics studies.

**Objective:**

This study aims to evaluate the metabolite profiles identified through metabolomics, as well as the associated pathways responsible for hyperglycemia during pregnancy and the postpartum period, in mothers with GDM compared with mothers without diabetes during pregnancy.

**Methods:**

We plan to recruit an equal number of patients with GDM and age- and BMI-matched mothers without GDM. Anthropometric data are collected at the first visit. Samples are collected at 3 time points: serum or plasma samples at the time of GDM diagnosis or at the first visit after diagnosis (24-28 weeks of gestation), placental and cord blood samples during delivery, and postnatal serum samples 4 to 12 weeks after delivery. Macroscopic and microscopic features of the placenta will be noted, and a histological score will be derived. Using liquid chromatography tandem mass spectrometry (LC-MS) with an untargeted approach, metabolite profiles will be identified in serum samples collected at diagnosis and postpartum, cord blood samples, and placental samples from both groups. The raw mass spectrometry (MS) data will be analyzed using the company-provided MS operator software. After data normalization, univariate analyses will be performed. Subsequently, all statistical analyses will be performed using MetaboAnalyst. Through these analyses, differences in the metabolite profiles and their pathways between mothers with and without GDM across pregnancy, starting from the diagnosis of GDM, will be identified. Data will be analyzed using R software. Clinical and demographic data will be presented as percentages, means, or medians, as appropriate. Logistic regression will be performed to assess the association between biomarkers and the placental histological score.

**Results:**

Ethics approval for the study was obtained from the Christian Medical College Vellore Ethics Committee, along with an institutional research grant in January 2025 and an Endocrine Society of Tamil Nadu grant in May 2026. Recruitment for the study began in June 2025 and was completed by June 2026; as of January 2026, a total of 35 patients had been recruited. The analysis of the samples using LC-MS, along with further statistical analysis, is expected to be completed in the first half of 2027.

**Conclusions:**

The findings from this study will provide insight into the metabolites or biomarkers, as well as the metabolic pathways involved, in the pathogenesis of GDM across the life course of mothers, compared with those of normoglycemic mothers, and will offer potential insight into the role of the placenta in GDM.

## Introduction

Gestational diabetes mellitus (GDM) typically manifests during pregnancy, typically in the second or third trimester, when diabetogenic placental hormone levels are at a peak. These hormonal changes occur to facilitate the growth of the developing fetus [1]. Globally, the prevalence of GDM is progressively increasing, with rates reaching up to 25% in some regions. The placental diabetogenic hormones, including human placental lactogen, growth hormone, and corticotropin-releasing hormone, play a crucial role in fetal growth. However, due to factors such as obesity, a mismatch may occur in which the pancreas fails to produce adequate insulin to meet the increased demands of pregnancy, further exacerbating insulin resistance and resulting in hyperglycemia, a hallmark of GDM.

Several metabolic pathways, including glycolytic pathways and fatty acid biosynthesis pathways, have been identified as being associated with GDM [2-4]. Several metabolic pathways that contribute to the development of diabetes have been identified through multiomics studies. Metabolomics can detect a broad spectrum of metabolic pathways by examining metabolites [5]. These include those pathways related to energy metabolism, such as the glycolytic pathway, the citric acid cycle, and oxidative phosphorylation, as well as lipid, amino acid, nucleotide, and 1-carbon metabolism. A meta-analysis showed that the levels of hexoses, branched-chain amino acids, aromatic amino acids, phospholipids, and triglycerides are associated with the development of type 2 diabetes (T2D) [6].

Hyperglycemia in GDM is generally less severe than that required for a diagnosis of T2D. Several amino acids, such as isoleucine, leucine, valine, alanine, and aspartate, as well as lipids such as lysophosphatidylcholine and palmitoylcarnitine, and carbohydrate metabolites, including pyruvate, lactate, 2-hydroxybutyrate, and 3-hydroxybutyrate, have been linked to an increased risk of GDM. However, glutamine, histidine, and sphingomyelin have been found to be associated with a reduced risk of GDM [7]. These metabolites are associated with pathways that contribute to hyperglycemia in diabetes. It has also been noted that there is a decrease in the levels of ecgonine methyl ester in placental tissues collected from patients with GDM [8]. This analysis also indicated that phospholipid metabolism was associated with the development of T2D based on maternal blood samples collected after birth [9]. All these studies have used plasma or serum from peripheral blood and examined associations between metabolite levels and GDM diagnosis.

Although several other mechanisms have been postulated, few studies have examined the pathogenesis of GDM throughout gestation, from diagnosis to the postpartum period [8]. Understanding the complex interplay of the placenta in GDM is crucial for elucidating the pathogenesis of hyperglycemia in pregnancy, which could help develop targeted interventions and improve outcomes for both mothers and children [8,10,11]. Studying metabolite profiles throughout pregnancy will help determine which metabolites are present at specific time points and whether they are uniquely present in mothers with GDM, potentially indicating a cause of GDM or serving as a diagnostic marker. Therefore, we aimed to evaluate metabolites and their associated pathways from pregnancy through the postpartum period in women with GDM, compared with those in normoglycemic mothers, using untargeted metabolomics.

## Methods

The primary aim of this study is to investigate metabolomic signatures and metabolites in maternal blood at the time of GDM diagnosis, in the placenta and cord blood, and in maternal blood at delivery and postnatally, compared with those in BMI- and age-matched normoglycemic mothers. We also plan to examine the macroscopic and microscopic features of the placenta, as well as their associations with specific metabolites or metabolic pathways.

### Ethical Considerations

The study is conducted in accordance with the ethical principles outlined in the Declaration of Helsinki. The institutional review board of Christian Medical College (CMC), Vellore, India, approved the protocol (2407109) on July 24, 2024. All study participants were informed about the study’s purpose, risks, and benefits and were told that participation was entirely voluntary. Written informed consent was obtained from all study participants after the study objectives were explained to them prior to participation. All data will be deidentified using a unique number before being sent for metabolomics analysis. No compensation will be provided because no intervention is involved in the study.

### Study Design and Setting

The study is an observational, prospective, case-control study that will be conducted among pregnant women attending the outpatient departments of endocrinology, diabetes and metabolism, and obstetrics and gynecology at CMC Vellore, India.

### Participants and Recruitment Criteria

We calculated the sample size using repeated measures (antenatal, placental, cord blood, and postnatal samples), with assumptions of an effect size (d=0.5), 4 time points with a correlation (*r*=0.6), 80% power, and α=.05. We required 25 patients in each group (GDM and non-GDM). Considering an attrition rate of up to 10% at each of 3 time points (antenatal, at delivery, and postnatal, including night deliveries), we plan to recruit up to 40 patients in the GDM and non-GDM groups.

So, we aim to recruit a maximum of 40 patients with GDM and 40 with normoglycemia from those attending outpatient clinics in the Department of Obstetrics and the Endocrinology, Diabetes, and Metabolism unit, with a required sample size of 40 participants in each group.

For this study, GDM is defined according to the International Association of Diabetes and Pregnancy Study Groups (IADPSG) diagnostic criteria. The study comprises 2 groups: a case group and a control group. The case group includes patients diagnosed with GDM, preferably women who are gravida 1 or 2, with no history of miscarriage, aged between 18 and 35 years, and with a BMI between 18.5 kg/m² and 30 kg/m² during the first trimester of pregnancy. Exclusion criteria include a history of miscarriage or recurrent abortions, hypertension or gestational hypertension, any medical illness, including morbid obesity or hypothyroidism, any past uterine surgery, or an assisted pregnancy. The control group consists of age- and BMI-matched healthy, normoglycemic pregnant women with normal results on a 75-gram oral glucose tolerance test (OGTT), with no history of comorbidities, and no use of medications affecting carbohydrate metabolism.

### Definition of GDM

All pregnant women diagnosed with GDM during routine OGTT screening at 24 to 28 weeks of gestation will be classified according to the IADPSG criteria: fasting glucose >92 mg/dL (5.1 mmol/L), 1-hour postload glucose ≥180 mg/dL (10.0 mmol/L), or 2-hour postload glucose ≥153 mg/dL (8.5 mmol/L). Women whose glucose levels are less than the IADPSG criteria are considered normoglycemic.

### Methodology of the Study

After written consent is obtained, participants are included in the study. Detailed demographic information and anthropometric measures are collected after consent is obtained. Blood samples, collected after an overnight fast, are taken from pregnant women with hyperglycemia and normoglycemic pregnant women during the first visit and stored for metabolomic analysis.

Following delivery, the placenta is collected for histopathological examination and metabolomic analysis. Cord blood is also collected for metabolomic analysis. Additionally, a comprehensive analysis of placental architecture, morphology, and histopathology will be conducted. Also, peripheral blood samples for a metabolomic study are collected from mothers during the 4 to 12 weeks postpartum period to investigate differences in metabolomic signatures.

This analysis will examine changes in placental histopathology and differences in metabolic signatures between women with and without GDM, as well as changes associated with delivery and the early postnatal period. Refer to Figure 1 for further details of the study workflow.

**Figure 1.**
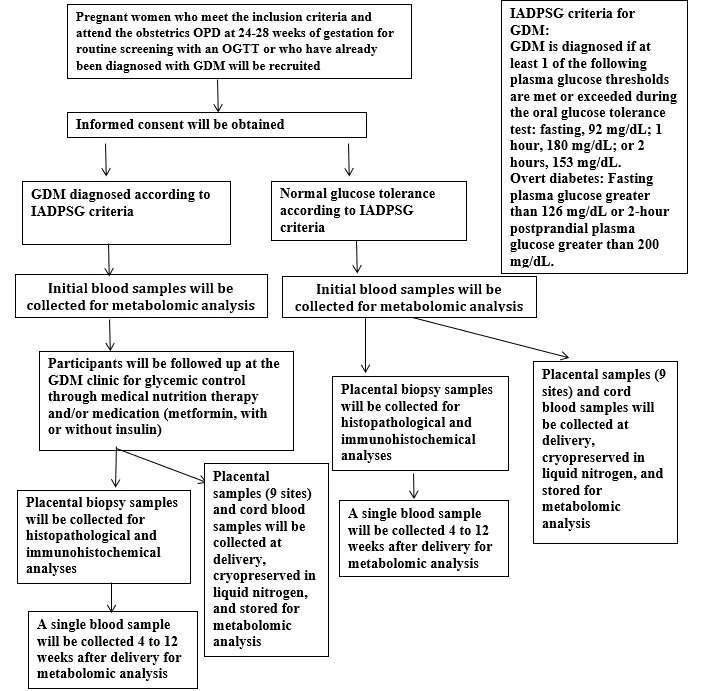
Patient recruitment workflow. GDM: gestational diabetes mellitus; IADPSG: International Association of Diabetes and Pregnancy Study Groups; OGTT: oral glucose tolerance test; OPD: outpatient department.

### Sample Collection Procedure and Storage

#### Blood Samples and Serum Preparation

All serum samples are labeled with a recognizable study number. We will collect whole blood in a tube using a vacutainer. After collection, the blood is allowed to clot without hemolysis by leaving it undisturbed at room temperature for up to 30 minutes. After the clot is removed by centrifugation at 1500 rpm for 10 minutes, the resulting supernatant will be designated as serum. Using a pipette, the serum is then transferred into small aliquots. The samples are being stored in a deep freezer at –80 °C until analysis. The samples will then be processed in batches, along with appropriate internal standards (ISs) and blanks, for metabolomics in accordance with the protocols.

#### Placental Sample Procedure

A major challenge in placental sample collection is the unpredictable timing of delivery during the third trimester and the mode of delivery, except in elective cesarean sections. Obtaining samples becomes difficult during nighttime deliveries or emergency cesarean sections. Placentas from morning deliveries will be used for metabolomics and histopathology, while those from nighttime deliveries will undergo histopathological examination only.

The placental samples are collected from 9 representative individuals, as described previously [12,13]. The sampling procedure is as follows. We collect the placenta within 30 minutes of delivery without placing it in saline or formalin. A photograph of the placenta is taken before sample collection. The samples are collected at equidistant points from the cord, avoiding the edges, fibrin deposits, infarcts, necrotic areas, and large blood vessels, with each piece measuring 1 to 2 cm in diameter. No additives are added to the placental tissue pieces. A total of 9 tissue pieces (3 fetal, 3 central, and 3 maternal) are placed into empty tubes and snap-frozen in liquid nitrogen. The tubes remain in liquid nitrogen for approximately 2 to 10 minutes. Afterward, the tubes are removed from liquid nitrogen and stored at –196 °C in a liquid nitrogen dewar until analysis. Photographs of the entire placenta are taken again, and the distance from the cord insertion site is measured to confirm the site of biopsy. The photographs are discarded after confirmation [8,14].

#### Cord Blood Sampling Method

Cord blood is collected immediately after the delivery of the placenta once the cord is clamped and cut from the placenta. The sample processing and storage procedures are the same as those followed during antenatal blood collection.

#### Pathology Assessments

All placentas will undergo comprehensive macroscopic and microscopic structural assessments [15]. Placental tissue will be assessed using the Amsterdam Placental Workshop Group (APWG) consensus classification, with maternal vascular malperfusion, fetal vascular malperfusion, and inflammatory lesions scored on a 0 to 3 scale for severity. GDM-specific features, including villous hypermaturity, trophoblastic hyperplasia, villous edema, and fibrinoid deposition, will also be assessed [16]. A comprehensive template for the macroscopic and microscopic evaluation of the placenta is provided in Table 1.

**Table 1.** Macroscopic and microscopic features of the placenta documented using the placental morphology template.

Categories and features	Findings
Macroscopic (gross) examination	
Placental morphology	Number: single or twin (monochorionic or dichorionic; monoamniotic or diamniotic) Shape: round, oval, bilobed, succenturiate, or battledore Measurements diameter: largest, smallest, and mean; radius (cm); surface area; thickness; volume; and weight
Placental membranes	Thin, smooth, shiny, thick, rough, translucent, opaque, complete, clear, colorless, meconium-stained, blood-stained, or amnion nodosum; hematoma (central or marginal); circummarginate or circumvallate (partial or complete)
Fetal surface	Smooth, rough, meconium-stained, or blood-stained
Maternal surface	Complete or incomplete; blood clots; cotyledons
Umbilical cord	Length (cm) and diameter (cm) Attachment: central, eccentric, marginal, or velamentous Pseudoknots, true knots, hypercoiling, torsion, strictures, constriction, or edema Blood vessel count: 2, 3, or 4 Thrombus: present or absent
Sections collected	Membrane, umbilical cord, fetal surface, and maternal surface
Microscopy	
Chorionic villi	Infarcts (%); fibrinoid necrosis (%); avascular villi; stromal fibrosis; chorangiosis; syncytial knots (%); basement membrane thickening; immature, edematous villi; and stromal hemosiderin
Intervillous spaces	Perivillous fibrinoid deposition (%), hemorrhage, and calcification
Decidua	Decidual vasculopathy or unremarkable findings
Placental membranes	Chorioamnionitis (acute or chronic), amnion nodosum, squamous metaplasia, and unremarkable findings
Umbilical cord	Normal blood vessels, thrombus, hemangioma, hematoma, and vasculitis

#### Data Storage and Reproducibility

All electronically documented study information will be stored in a dedicated, secure, and restricted-access REDCap (Vanderbilt University) database. Only study investigators and key study staff will have access to the database.

### Statistical Analysis

#### Data Entry and Descriptive Statistics

All data entry and cleaning will be conducted by data entry staff using a double-entry verification process. All analyses will be performed using R (version 26; R Project for Statistical Computing). Descriptive statistics will summarize participant characteristics. All continuous variables will be checked for normality before analysis. All clinical characteristics and demographic data will be presented as either categorical data (percentages) or continuous data (means or medians, as appropriate). We will include all the data for final analysis even if not all the samples were obtained, following the intention-to-treat analysis.

#### Metabolomics Assessment

All serum (antenatal, cord blood, and postnatal serums) and placental samples will undergo untargeted metabolomic profiling to identify hundreds of metabolites spanning carbohydrate, lipid, amino acid, and energy metabolism pathways. Metabolite profiling will be performed using liquid chromatography tandem mass spectrometry (LC-MS).

The acquired LC-MS or raw mass spectrometry (MS) data will initially be processed using Agilent MassHunter (Agilent Technologies Inc) software for data quality assessment, chromatographic inspection, and peak detection. The resulting data files will then be processed using MS-DIAL (version 5.5; RIKEN Center for Sustainable Resource Science; CompMS; MS-DIAL software program for untargeted metabolomics) for peak detection, noise filtering, chromatographic peak alignment, deconvolution of MS or MS spectra, and metabolite annotation. During MS-DIAL processing, appropriate parameters will be applied for mass detection, peak height threshold, minimum peak width, MS1 or MS2 tolerance, retention time tolerance, and chromatographic peak alignment based on the LC-MS or MS acquisition conditions. For metabolite annotation, accurate precursor mass and MS or MS spectral matching will be used against available reference libraries, with isotope pattern and retention time information considered where available.

Quality control (QC) samples will be prepared by pooling representative samples and analyzed after every 10 study samples throughout the analytical sequence to monitor instrument performance, signal stability, analytical reproducibility, and potential signal drift. Blank samples will also be included to identify background signals and contaminants originating from solvents, sample preparation, or the analytical system. Features with substantial contributions from blank samples will be removed during blank filtering prior to downstream analysis.

ISs or spiking controls will be added to the sample extraction protocol to monitor extraction efficiency and analytical variability. Following initial QC and filtering, metabolite intensities will be subject to IS-based normalization to correct for extraction-related and instrumental variability. Appropriate ISs will be used to normalize the corresponding metabolite signals. Following IS normalization, the data will be further processed using median-based normalization to minimize the remaining sample-to-sample variation.

Metabolite annotation will be performed based on a combination of accurate precursor mass, retention time, isotope pattern, and MS or MS spectral matching against available reference spectral libraries. The resulting quality-controlled and normalized metabolite dataset will subsequently be used for statistical analysis and biological interpretation, including pathway analysis using appropriate metabolomics bioinformatics tools.

The distribution of metabolite abundances will subsequently be evaluated using medians and IQRs, particularly because metabolomics data may not follow a normal distribution and can contain extreme values or outliers. The normalized metabolomics data will then be log-transformed to reduce right-skewness and stabilize variance.

For the statistical analyses, given the high dimensionality of metabolomics data and the large number of metabolites evaluated simultaneously, correction for multiple hypothesis testing is essential to reduce the risk of false-positive findings. We will further expand the statistical analysis section of the manuscript to explicitly describe our multiple-testing strategy.

For 2-group comparisons, Student *t* test or the Mann-Whitney *U* test will be used as appropriate, while ANOVA or Kruskal-Wallis tests will be used for comparisons involving more than 2 groups. The resulting metabolite-wise nominal *P* values will be subjected to Benjamini-Hochberg false discovery rate (FDR) correction across the corresponding set of simultaneously tested metabolites. The resulting FDR-adjusted *P* values will be used to determine statistical significance, with an adjusted *P* value of <.05 considered significant.

In addition to statistical significance, metabolite-level effect sizes will be considered to assess the magnitude of biological changes. Fold changes and/or log_2_ fold changes will be calculated and reported alongside nominal *P* values and FDR-adjusted q-values. Thus, the interpretation of differential metabolites will be based on both the statistical evidence and the magnitude of the observed metabolic alteration.

For correlation analyses involving multiple metabolites and clinical or biochemical variables, Spearman correlation coefficients will be calculated, and the resulting *P* values will similarly be corrected using the Benjamini-Hochberg FDR procedure. This will ensure that the increased probability of false-positive findings arising from multiple correlation tests is appropriately controlled.

Annotated metabolites will subsequently be used for biological interpretation and pathway analysis. KEGG (Kyoto Encyclopedia of Genes and Genomes) will be used for metabolic pathway mapping, while MetaboAnalyst (version 6) will be used for pathway analysis and visualization [16,17,18].

The metabolites and their pathways that are significant at different time points (antenatal, placental, cord blood, and postnatal time points) will be tabulated and correlated to determine whether any metabolites or their pathways differ at a specific time point. Moreover, metabolites and their pathways identified in the placenta will be correlated with microscopic architecture scores from the APWG and with GDM-specific changes in the placenta using Spearman rank correlation. Univariate and multivariate logistic regression analyses will also be performed to assess the association between the microscopic architecture score and the identified metabolites. To compare the 2 groups, the chi-square test or an independent-samples *t* test will be used, with *P*<.05 considered significant.

## Results

The first participant was recruited for the study in June 2025 from the Department of Endocrinology, Diabetes & Metabolism and Obstetrics and Gynaecology at CMC, Vellore, India. At the time of manuscript submission, 35 participants had been recruited, and placental sections from 21 participants were available for metabolomics analysis. Recruitment was expected to be completed in June 2026. Metabolomic analyses are expected to be completed in the first half of 2027. The study results will be reported in accordance with the STROBE (Strengthening the Reporting of Observational Studies in Epidemiology) guidelines for observational studies (Checklist 1). The flowchart showing participant recruitment and sample collection is presented in Figure 1.

## Discussion

### Anticipated Findings

The literature indicates that certain biomarkers identified through metabolomics are unique to GDM diagnosis. However, no studies have evaluated changes in metabolomic profiles throughout pregnancy and the postnatal period.

Numerous studies have examined several biomarkers to identify or predict GDM or early onset of future diabetes. For instance, in peripheral blood, levels of arachidonic acid and linolenic acid were found to be significantly elevated in mothers with GDM compared with those without GDM [19]. Furthermore, studies have shown that metabolites from fatty acid oxidation and purine metabolism are abundant in placental tissue from mothers with GDM. In particular, levels of omega-6 fatty acids, such as linoleic acid, arachidic acid, and palmitic acid, as well as omega-3 fatty acids, were depressed [8]. Butanoate metabolism was found to be enriched in the uncontrolled hyperglycemia group, and increased L-leucine levels were noticed in the uncontrolled hyperglycemia group compared to the nondiabetic group [20]. Several pathways have been found to be associated with GDM. However, none of these pathways has been studied across the course of pregnancy and the postnatal period, from diagnosis to the postnatal period.

Therefore, there is a need for an expanded analysis of blood and placental samples, including postnatal blood samples, through molecular techniques such as metabolomics and metabolic network analysis, to provide a comprehensive understanding of metabolic alterations associated with GDM and its course throughout pregnancy [10,20,21]. Moreover, our study may enable the identification of a wide range of metabolites, potentially revealing novel biomarkers and metabolic pathways associated with the pathophysiology of GDM throughout pregnancy and the postnatal period, which could be used to predict GDM.

Second, by comparing the metabolic profiles of mothers with GDM with those of normoglycemic mothers, we can find specific changes that could contribute to the development or progression of future diabetes among mothers with GDM [22-24]. Moreover, pathway analysis will help identify potential mechanisms underlying newer oral antidiabetic agents.

The strength of our study lies in its unique focus on metabolic changes across pregnancy and the postnatal period, encompassing the placenta and cord blood. A limitation is that, due to the complexity of the research, we plan to recruit a relatively small sample comprising 25 patients with GDM and 25 with normoglycemia, for a total of 50 patients, with all 3 time point blood samples collected at all 3 time points and cord blood samples collected at delivery. We do not have samples from the first trimester or from before the diagnosis of GDM.

Study outcomes will be disseminated through presentations at scientific conferences and publication in peer-reviewed journals.

### Conclusions

Through our study, we aim to elucidate the metabolic pathways that change during pregnancy in women with GDM, compared to those in normoglycemic controls. We anticipate that metabolites and their associated pathways may be identified across the period from diagnosis through the postnatal period, which could help predict GDM or deepen our understanding of its pathogenesis.

The study is likely to provide additional information regarding the placenta and its role in the pathogenesis of diabetes during pregnancy among mothers with GDM and may assist in the development of new therapeutic options for women with GDM.

## Supplementary material

10.2196/92064 Checklist 1 STROBE checklist.

## Abbreviations

APWG: Amsterdam Placental Workshop Group
CMC: Christian Medical College
FDR: false discovery rate
GDM: gestational diabetes mellitus
IADPSG: International Association of Diabetes and Pregnancy Study Groups
IS: internal standard
KEGG: Kyoto Encyclopedia of Genes and Genomes
LC-MS: liquid chromatography tandem mass spectrometry
MS: mass spectrometry
OGTT: oral glucose tolerance test
QC: quality control
STROBE: Strengthening the Reporting of Observational Studies in Epidemiology
T2D: type 2 diabetes
